# Attendance of Medical Lectures in London

**Published:** 1859-08

**Authors:** 


### THE ECRASEUR.

The *Lancet* says "the ecraseur has become almost obsolete in London hospital practice. We have ever looked upon its use as an unsurgical proceeding. Like every other novelty, it has had its trial, and it will soon be altogether laid aside, unless in some very exceptional surgical maladies, in which the risk of hemorrhage may again require its aid."

---

### PROFESSIONAL HABITS IN 1670.

The great Sydenham, in the last treatise which he wrote, gives the following account of his own manner of living:

"In the morning when I rise, I drink a dish or two of tea, and then ride in my coach till noon; when I return, I moderately refresh myself with any sort of meat of easy digestion that I like (for moderation is necessary above all things); I drink somewhat more than a quarter of a pint of Canary wine, immediately after dinner every day, to promote the digestion of food in my stomach, and to drive the gout from my bowels. When I have dined I betake myself to my coach again, and when my business will permit, I ride into the country two or three miles for good air. A draught of small beer is to me instead of a supper, and I take another draught when I am in bed, and about to compose myself to sleep."

---

### ATTENDANCE ON MEDICAL LECTURES IN LONDON.

We have become so accustomed to hearing the names of distinguished teachers in the various departments of medicine and surgery in London, that we fancy that large numbers of students and practitioners will avail themselves of this privilege of hearing them.

On this point Dr. A. B. Palmer, senior editor of the *Peninsular and Independent Medical Journal*, writes as follows from London to that journal:

Dr. Walshe has delivered only one set clinical lecture this term, which one I had the pleasure of hearing. It was upon mediastinal tumors, and based upon a case which occurred some time before. It was an exceedingly able lecture, philosophical and discriminative—analyzing closely all the symptoms, comparing them with such as might have been produced by other pathological conditions, and with which they may have been confounded, etc.; and it was delivered in the clinical lecture room to just *thirteen* students, two of whom were asleep during most of the hour. The places of these sleeping ones were supplied by another American physician and myself, so that there was still an audience of a baker's dozen. I have been astonished everywhere to find the classes listening to lectures, so small.

There is only a dozen attending Dr. Jenner's lectures, on pathological anatomy; some fifteen or sixteen Mr. Erichsen's clinical lecture, which I heard; and in the large school and hospital of St. Bartholomew, the classical Dr. West, in his regular course on obstetrics, is lecturing to between thirty and forty; and Dr. Murphy, in the same regular course, at University College, is addressing about half that number. The largest class I have seen assembled for a lecture was at Dr. Garrod's, at University College, and that was between fifty and sixty. The reason of this is to be found in the large number of schools, and the more moderate number of students studying in the Metropolis. The practice of lecturing to so few gets the professors in the habit of being dull in their manner. As a body, they are much more prosy than those I have been accustomed to hear in our own country.

Dr. Garrod is an active and industrious man, and is on duty in the hospital. He has fewer students attending in his ward than Dr. Walshe. I think I have not seen present more than half a dozen, and the average of those attending the physicians in their wards in all the hospitals, is but little over this number. The surgeons are generally better attended, their numbers being from ten to twenty-five, at most, except when there are operations in the amphitheatres.

---

THE Legislature of New York has incorporated a Preparatory School of Medicine in the city of New York. The following are the Lecturers:—on Surgery, John O. Bronson, M.D.; Midwifery and Diseases of Women and Children, Chas. A. Budd, M.D.; Chemistry and Toxicology, Bern L. Budd, M.D.; Legal Medicine, Hon. John H. Anthon; Physiology and Micrology, Charles K. Briddon, M.D.; Botany and Materia Medica, Geo. Thurber, M.D.; General and Special Pathology, Geo. A. Quimby, M.D.

The Faculty are empowered, under certain restrictions, to confer the degree of *Bachelor of Medicine.*

